# A Novel Intraoral Mandibular Osteotomy For Set-Back Surgery In Complex Mandibular Anatomy

**DOI:** 10.1007/s00784-025-06239-y

**Published:** 2025-03-28

**Authors:** Mohammed Omara, Sherif Ali, Yassin Salah Alian, Nehal Ibrahim Shobair

**Affiliations:** 1 Oral and Maxillofacial Surgery Department, Faculty of Dentistry, Cairo University, 11 Saraya Street, Manial, Cairo Egypt; 2 Oral and Maxillofacial Prosthodontics Department, Faculty of Dentistry, Ain Shams University, Cairo, Egypt; 3 Oral and Maxillofacial surgery Department, Misr International University, Cairo, Egypt

**Keywords:** Orthognathic surgery, Angle class III, Inferior alveolar nerve injuries, Surgery, computer-assisted, Prognathism, Intraoperative complications

## Abstract

**Objective:**

Complex mandibular anatomy including rolled-out inferior mandibular border, thin rami with cortically adherent inferior alveolar nerve (IAN) complicate the application of the current mandibular osteotomies. This study aims to introduce an intraoral Inverted-L Ramus Osteotomy (ILRO) modified with IAN lateralization and intra-canal osteotomy for management of complex mandibular anatomical variations during mandibular setback surgery.

**Patients and Methods:**

This prospective study included 20 skeletal class III patients (mean age: 21.6 ± 3.3 years) with complex mandibular anatomy indicated for mandibular setback surgery (mean setback: 6.05 ± 1.1 mm). Preoperative CBCT imaging, digital planning, and fabrication of cutting / drilling guides were performed. Surgery involved mandibular setback through the application of the ILRO modified with nerve lateralization and intra-canal osteotomy. IAN function evaluated preoperatively at intervals up to one year postoperatively. Data on bad splits and surgical duration were also analyzed.

**Results:**

The mean surgical duration was 2.26 ± 0.21 h, with bilateral IAN exposure completed in 11 ± 3.2 min per side. All 40 osteotomy sites were separated without bad splits. Neurosensory deficits were observed in 90% of patients at two months, decreasing to 35% at six months and 5% at one year.

**Conclusion:**

The introduced osteotomy overcomes the limitations of the traditional mandibular osteotomies in dealing with mandibular complex anatomy with adequate IAN protection and split segments integrity during mandibular setback surgery.

**Clinical relevance:**

The introduced osteotomy provides a safe alternative to the current mandibular osteotomies utilized in mandibular setback surgery.

## Introduction

Mandibular setback surgery is a complex process when challenged by anatomical variations such as rolled-out inferior mandibular border, thin rami with cortically adherent inferior alveolar nerve (IAN) and inferiorly seated IAN canal [1]. Traditional jaw osteotomies, including Bilateral Sagittal Split Osteotomy (BSSO), Vertical Ramus Osteotomy (VRO) and the traditional Inverted-L Ramus Osteotomy (ILRO), have been widely used to correct mandibular deformities [2–4]. However, these techniques are limited in cases with complex anatomical variations, as they pose risks of unfavorable splits, increased nerve injury, and extensive distal interferences during setback movements [5–7]. 

BSSO is the workhorse for correction of different mandibular deformities owing to its versatility [2]. It also allows for rigid internal fixation with maximum bony contact. Nevertheless, Mandibular anatomical variations such as thin rami, low marrow to cortex ratio with cortically adherent IAN and rolled out inferior mandibular border are considered as major limitations for this osteotomy. These variations complicate the splitting procedure and increase the risk for bad split and IAN injury [1, 6]. VRO is an established method in the management of mandibular prognathism with minimal risk of bad splits and IAN injury. However, it doesn’t allow for rigid fixation that necessitates prolonged maxilla-mandibular fixation. Moreover, the massive distal interferences in setback cases hinder its applicability [3]. 

The traditional ILRO is a competent alternative to the BSSO in terms of versatility and amenability to rigid fixation with a lower risk of bad splits and neurosensory disturbance. Despite its advantages, ILRO is performed via an extraoral approach, and it faces limitations in mandibular setback cases due to distal interferences and the need for IAN protection [4, 8, 9]. Subsequently, the C-osteotomy introduced by Caldwell et al. 1968, modified the traditional ILRO to be performed via an intraoral approach by extending the long descending arm anteriorly below the IAN canal, this anterior arm is usually thin and prone to unfavorable splits with neurosensory disturbances, especially in cases with inferiorly positioned IAN canals [8–10]. 

Recently, Senior et al. modified the C-osteotomy’s anterior extension to be performed at the level of the inferior border of the IAN canal while protecting the IAN upward to run as a sagittal split osteotomy instead of bicortical traditional anterior extension. This modification possesses the same limitations of the traditional BSSO [10]. These limitations highlight the need for an innovative technique that combines safety, versatility, and effectiveness in managing complex mandibular anatomical variations. Specially that the advances in digital planning and customized surgical approaches have offered solutions to some of these limitations. This study aims to introduce a modified intraoral ILRO involving IAN lateralization and intra-canal osteotomy for management of complex mandibular anatomical variations with IAN protection and preservation of split segments integrity during mandibular setback surgery.

## Patients and methods

**Fig. 1:**
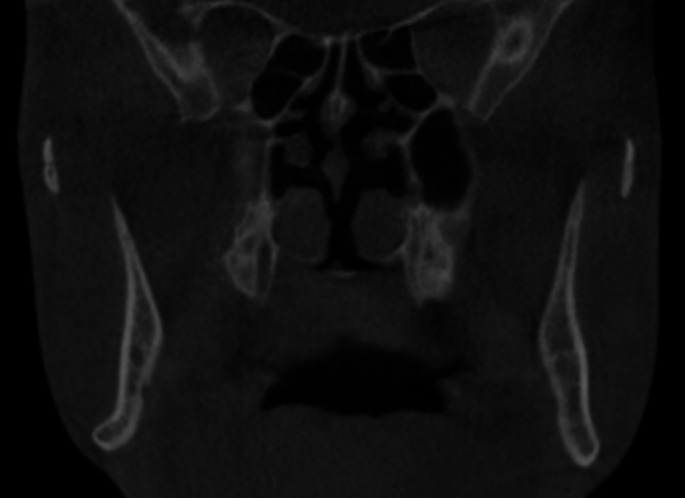
A CBCT coronal cut showing the thin mandibular rami, rolling out of the inferior mandibular border, and the IAN proximity to the buccal cortex

**Fig. 2:**
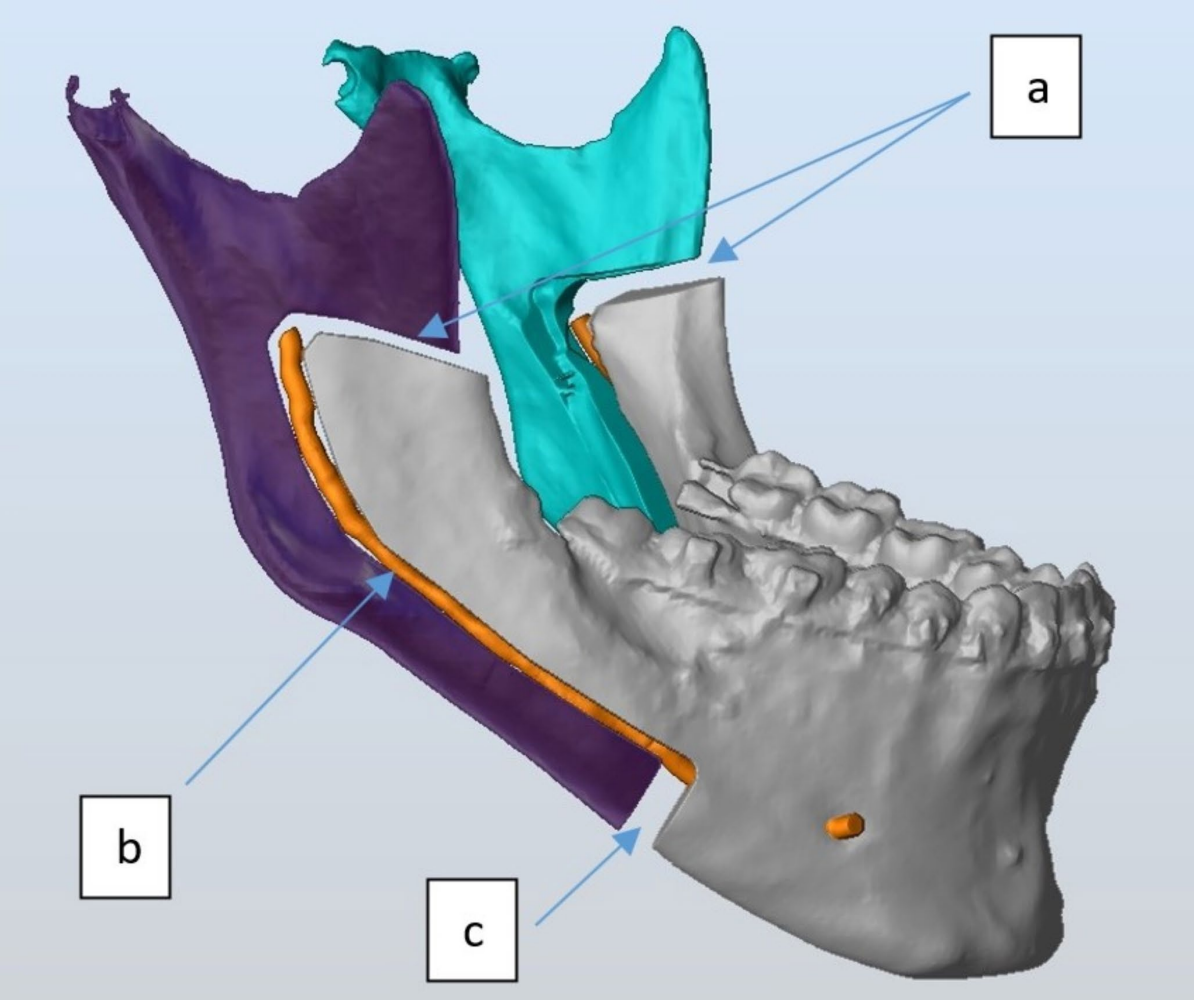
A snapshot showing the current osteotomy design: **a**; The horizontal Ramal cut extending from the anterior border of the ramus above the level of the lingula and just posterior to it. **b**; A lateral intracanal osteotomy that starts from the most posterior end of the horizontal cut and then curves down to path through the mandibular canal. **c**; The Two vertical cuts extend from the most anterior end of the intracanal osteotomy to the inferior border of the mandible

The study was registered on ClinicalTrials.gov under the registry number NCT05397002. It adhered to the Declaration of Helsinki on medical research ethics and received approval from the Institutional Research Ethics Committee of the Faculty of Dentistry, Cairo University (IRB number: 151020). This prospective, single-center case series included 20 patients consecutively recruited from the outpatient clinic of the Department of Oral and Maxillofacial Surgery, Faculty of Dentistry, Cairo University. Patient recruitment began in October 2020 and continued until April 2022. The intervention phase was conducted from December 2020 to April 2022, with follow-up extending from December 2020 to April 2023. Data collection was carried out throughout the study duration, concluding in May 2023. Patients were selected according to the following inclusion criteria: (1) Adult patients complaining of skeletal class III malocclusion requiring mono-maxillary or bi-maxillary surgical intervention; (2) CBCT examination revealed anatomical hindrances that interfere with the application of BSSO indicating the application of the currently modified intraoral ILRO osteotomy (thin mandibular rami with minimal or no medullary bone, inferior alveolar nerve proximity to the buccal cortex along the length of the BSSO cuts, lateral bending of the inferior mandibular border at molar angle region, high mandibular foramen, inferiorly seated IAN canal and extensive distal interferences) (Fig. 1) and (Fig. 2). Patients with previous extensive jaw surgery, mandibular pathological lesions, or temporomandibular joint dysfunction were excluded from the study.

### Preoperative preparation and virtual planning

A thorough clinical examination was performed; preoperative photographs and plaster dental cast models were obtained for all enrolled patients. Selected patients were submitted to the orthodontic department for leveling, alignments, and decompensation of dental arches. After orthodontic preparation upper and lower impressions were obtained to fabricate a stone model and generate a virtual model via optical scanning. Using CBCT machine (Scanora^®^ 3D with AutoSwitchTM, Soredex, Helsinki, Finland - Exposure parameters were 85 kVp, 15 Ma, and 6 cm FOV) a full-skull CBCT image was captured in a natural head position with the patient slightly biting and with all the facial muscles at rest. The CBCT was used along with the virtual models to construct the virtual skull model for diagnosis, digital planning, guide designing, and manufacturing.

**Fig. 3:**
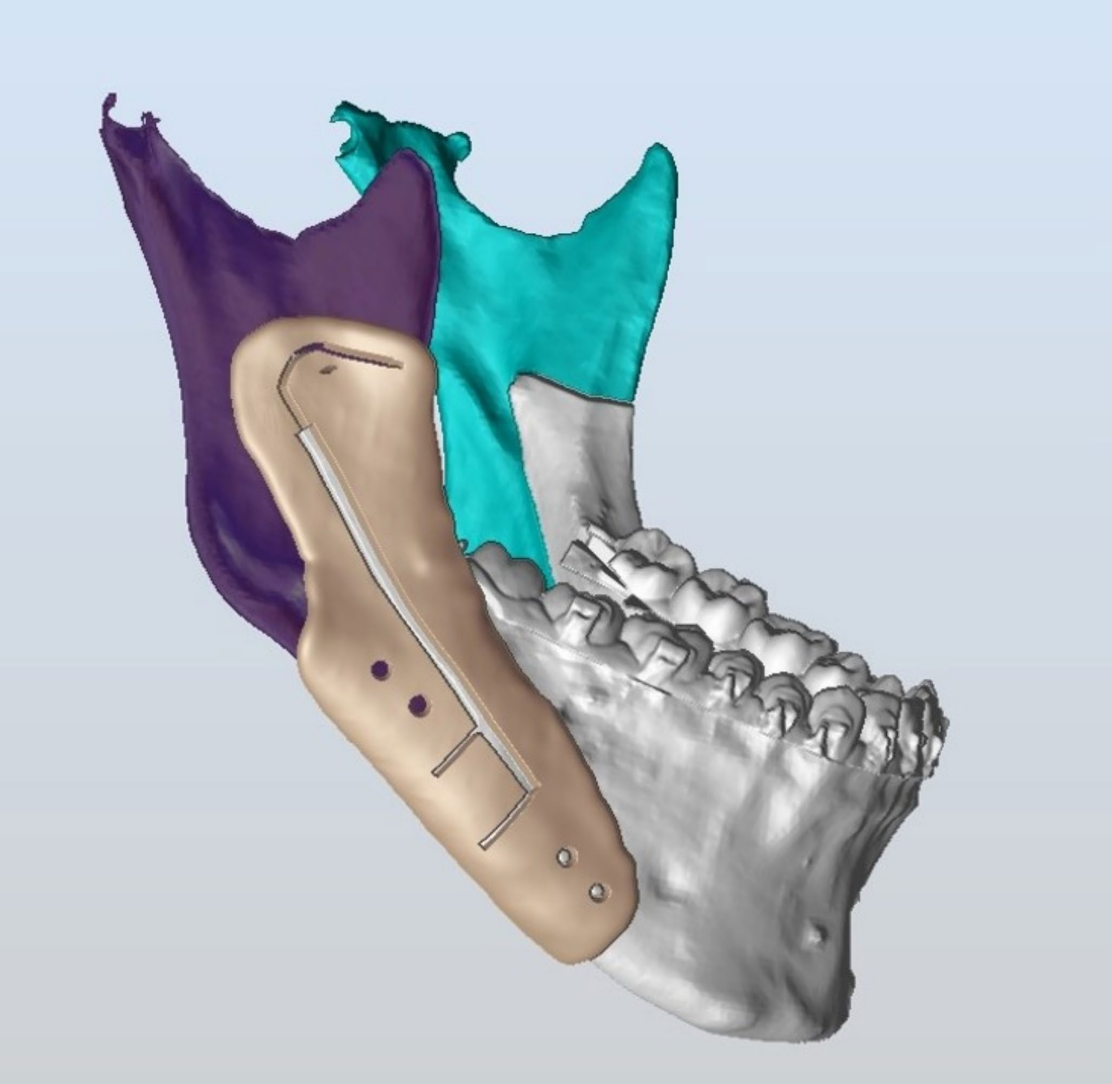
A snapshot showing the 3D virtual design of the cutting and drilling guide

DICOM files were imported to the planning software ProPlan CMF software (Materialise, Belgium). Using the software, mandibular bone morphology was 3D analyzed to assure their correspondence with eligibility criteria. IAN was segmented, traced, and marked along the lateral surface of the mandibular canal. Osteotomy lines were planned to consist of Four cuts: (1) A horizontal ramal cut extending from the anterior border of the ramus above the level of the lingula and just posterior to it. (2) A lateral intra-canal osteotomy that starts from the most posterior end of the horizontal cut and then curves down to path through the mandibular canal. (3) Two vertical cuts extend from the most distal end of the intra-canal osteotomy to the inferior border of the mandible. The created bone segment between the two vertical cuts represents the amount of mandibular setback (Fig. 2). A corrected 3D model was fabricated to bend the plates and identify the screw holes. Then the model with the identified screw holes was scanned, and the cutting and drilling guides were fabricated using a PLA resin and a DLP 3D printer [11]. The guide was designed with a slit, holes, and central trough to landmark the osteotomy lines, screw holes’ position, and nerve path respectively (Fig. 3). Post-printing guides processing, and cold sterilization was performed.

### Surgical procedures

**Fig. 4:**
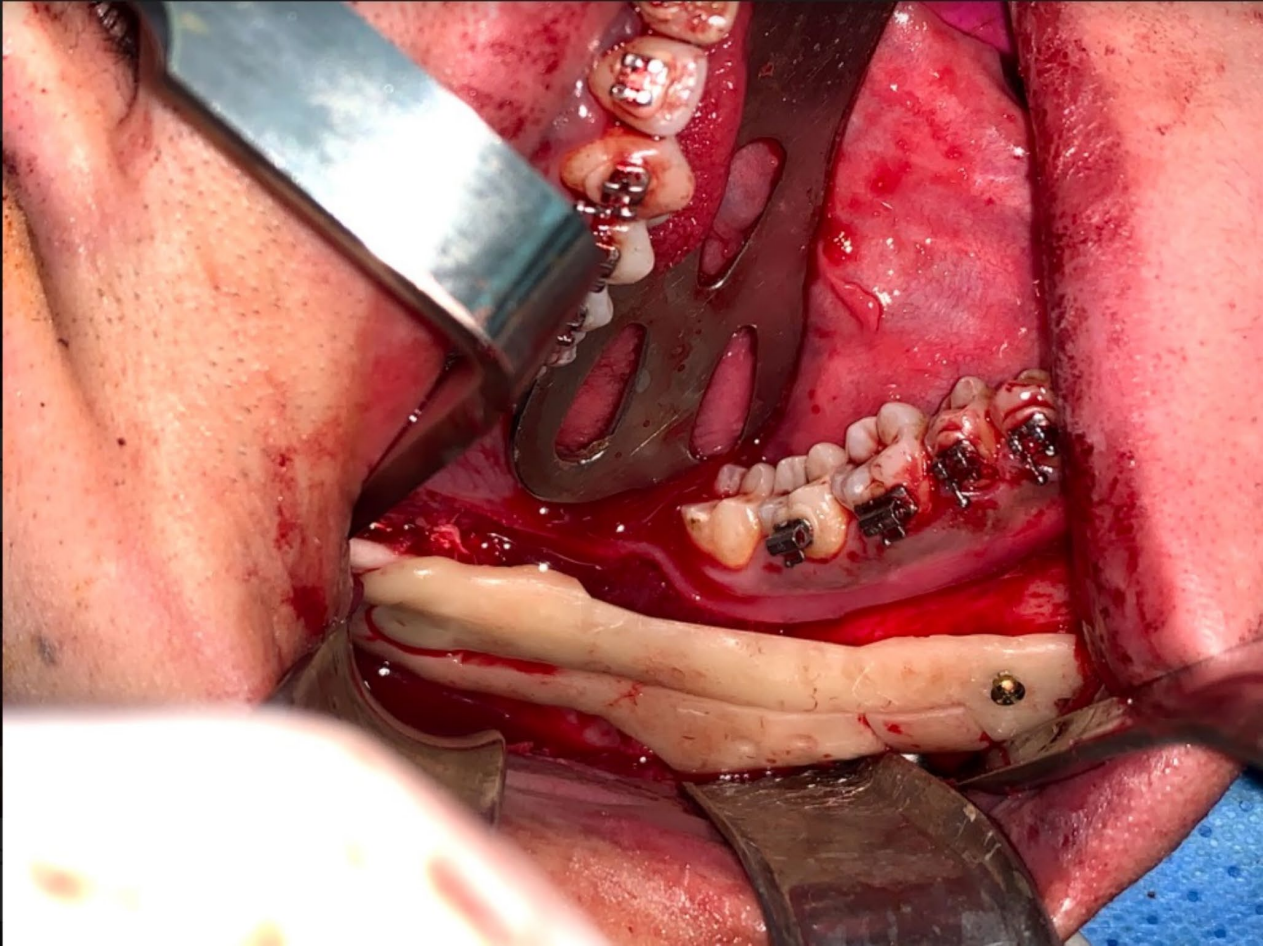
Intraoperative photograph showing the guide seated in place

**Fig. 5:**
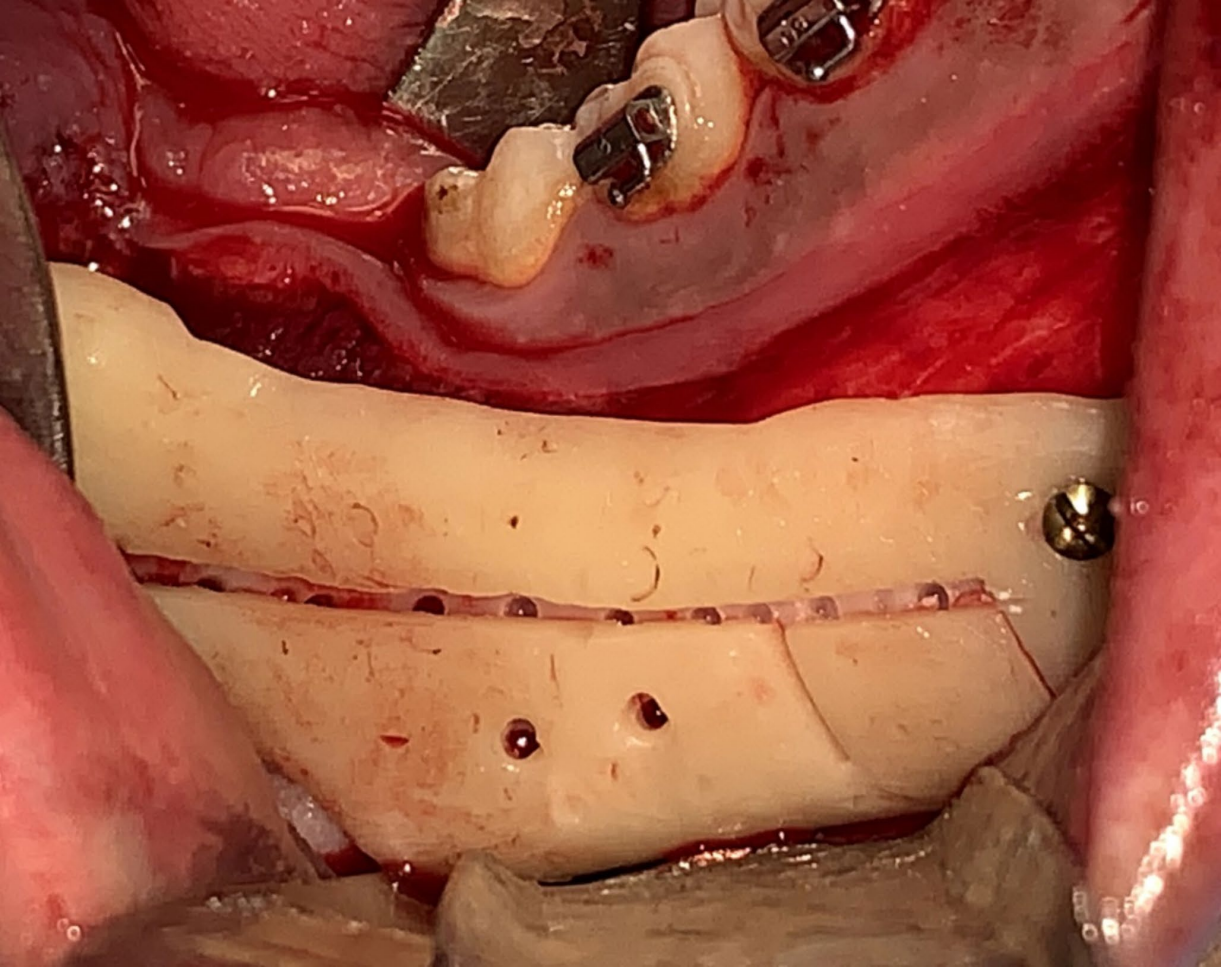
Intraoperative photograph showing the marking holes for IAN course tracing

**Fig. 6:**
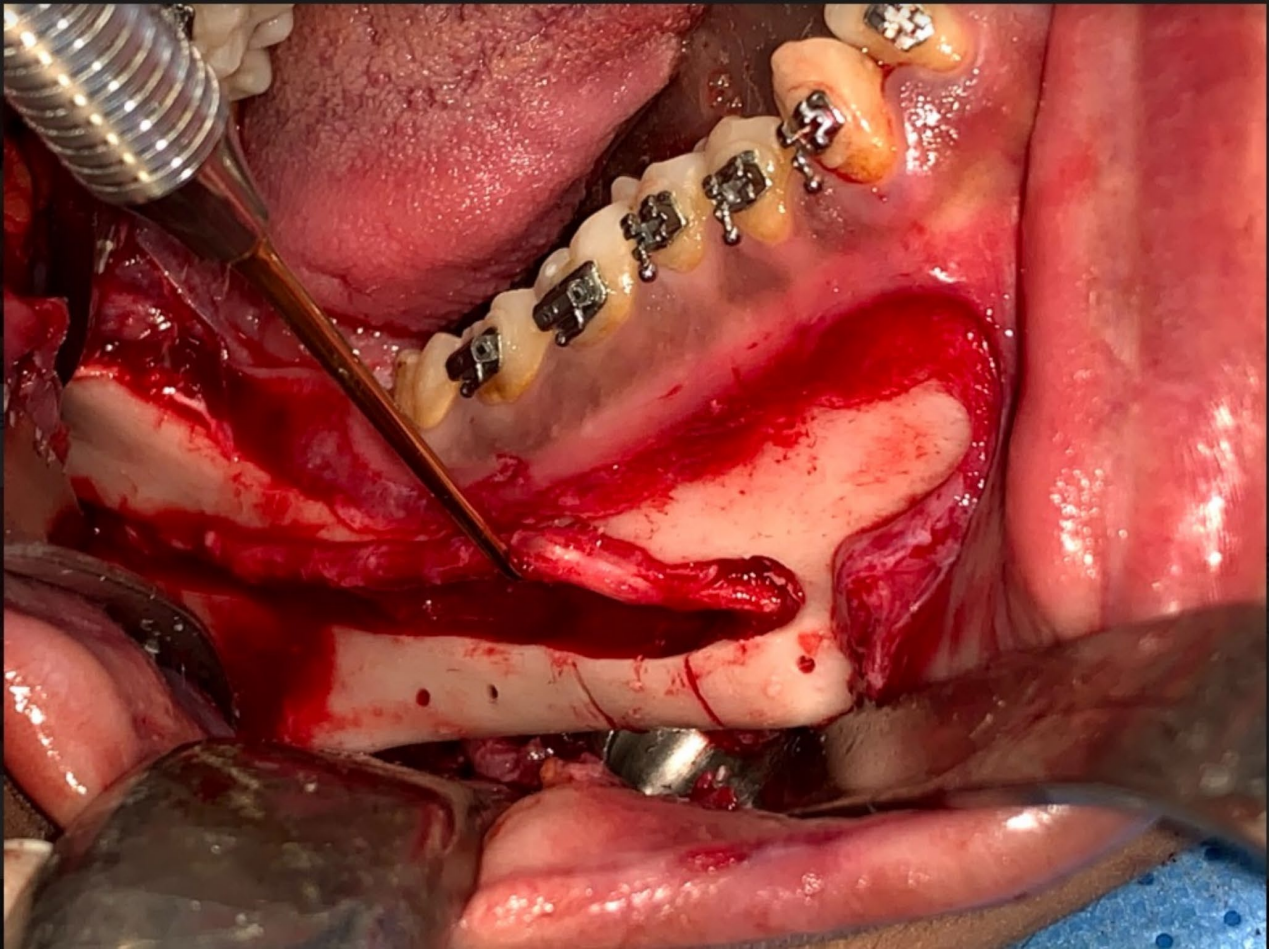
Intraoperative photograph showing the application of the designed osteotomy lines and drill holes: Inferior alveolar nerve lateralization and intracanal osteotomy through the lateral ostectomy, two vertical osteotomy markings revealed the amount of setback and the drill holes’ location for pre-bent plate guidance

**Fig. 7:**
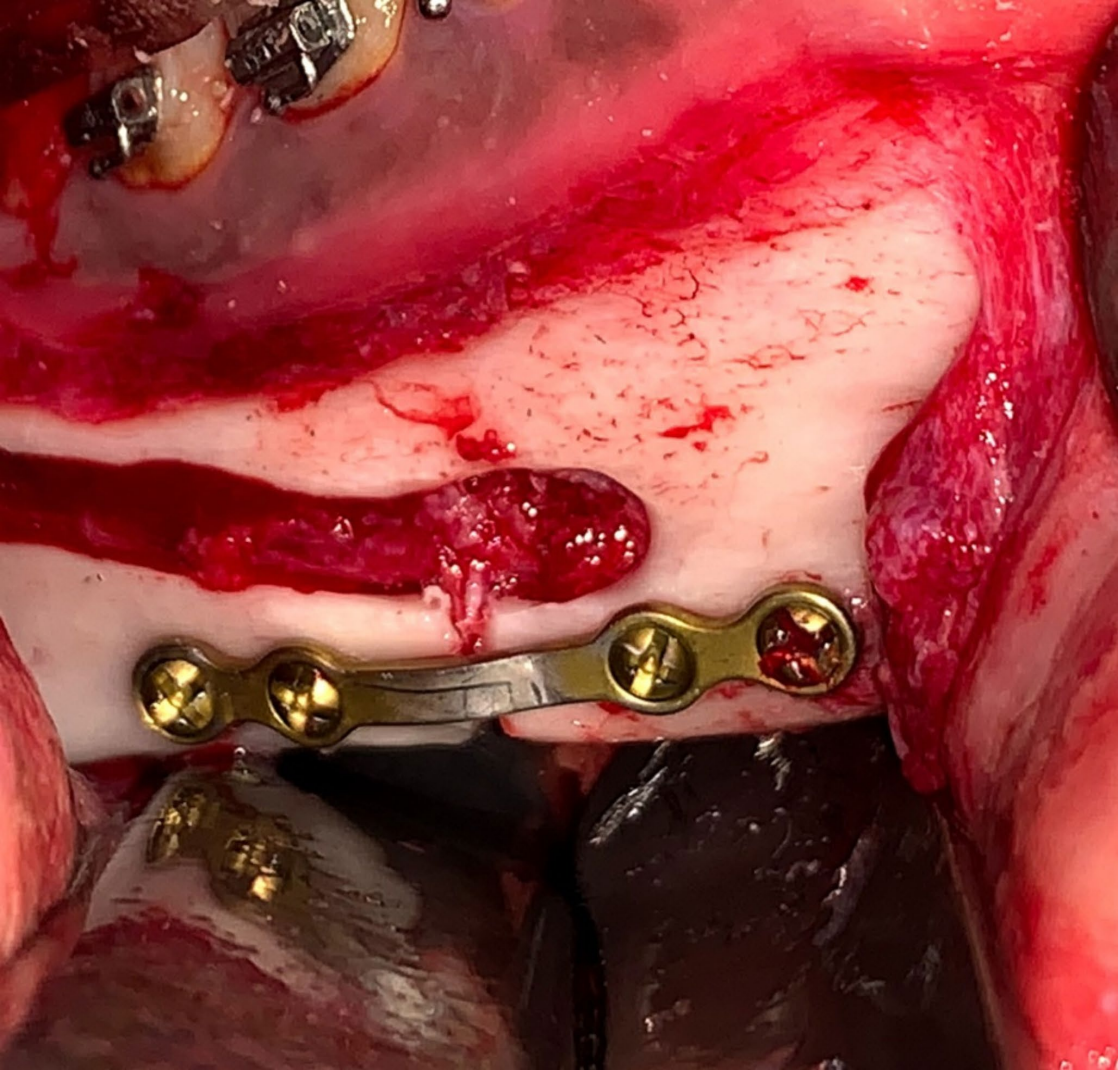
Intraoperative photograph showing: Mandibular setback and pre-bent plate osteosynthesis

Based on the preoperative clinical and radiographic assessment and virtual planning enrolled skeletal class III patients were categorized in to patients requiring mono-maxillary or bimaxillary surgical intervention. The surgical procedure was performed under general anesthesia. In Bi-maxillary cases, the maxillary surgery was performed first, segment was mobilized and positioned using a cutting and drilling surgical guide associated with pre-bent plates osteosynthesis, then the mandibular surgery was performed. The mandibular surgery starts with bilateral intraoral mandibular incisions. The surgical guide was placed in position (Fig. 4). Screw holes and osteotomy lines were performed. A reference marking points were performed using a surgical fissure bur through the nerve tracing central trough of the surgical guide to mark the nerve pathway (Fig. 5). The device was removed, and the marked pathway was followed using a sinus lifting round stone to uncover the lateral cortical bone overlying the nerve. The nerve was then carefully repositioned in a lateral and upward direction using a nerve hook (Fig. 6). The intracanal osteotomy was completed through the uncovered mandibular canal using a reciprocating saw to connect the upward horizontal ramus cut with the two vertical inferior border cuts. The osteotomy lines were confirmed using spatula chisels then the proximal and distal segments were successfully separated. The distal interferences were removed while the IAN was safely lateralized, and the mandibular segments were repositioned guided with the pre-bent plates and screw holes (Fig. 7).

### Neurosensory test procedure

The IAN function was examined preoperatively, one week, two months, six months, and one year postoperatively. A light brush technique and two-point discrimination were used at each follow-up consultation. Anesthesia was evaluated using the pin-prick test. The altered sensation was categorized as either hypoesthesia or as “positive” sensory phenomena such as hyperesthesia, dysesthesia, or paranesthesia. The location of the sensory impairment was categorized as the left lower lip, and right lower lip.

### Statistical analysis

It was performed using SPSS (Statistical package for the social sciences- IBM^®^ SPSS^®^ Statistics Version 20 for Windows, IBM Corp., Armonk, NY, USA). Quantitative data were represented as mean ± standard deviation. Qualitative data were represented as percentage or frequency.

## Results

This case series included 20 patients (mean age: 21.6 ± 3.3 years; 13 females, 7 males) with skeletal class III malocclusion requiring mandibular setback surgery. 14 patients (70%) underwent bimaxillary surgery, while 6 patients (30%) underwent mono-maxillary surgery. The mean magnitude of the mandibular setback was 6.05 ± 1.1 mm. The average duration of the mandibular procedure, from the initial incision to suturing, was 2.26 ± 0.21 h. Inferior Alveolar Nerve (IAN) exposure was successfully achieved bilaterally within 11 ± 3.2 min per side (Table 1).

**Table 1:** Demographic and Surgical characteristics of patients undergoing mandibular setback surgery

Case no.	Age	Gender	Type of the operation	Magnitude of mandibular setback (mm)	Time for mandibular operation (hrs.)
1	19	Male	Bimaxillary	6	2.4
2	25	Female	Monomaxillary	5	2.2
3	21	Female	Bimaxillary	7	2
4	18	Female	Bimaxillary	5	2
5	20	Female	Bimaxillary	8	2.4
6	23	Male	Bimaxillary	6	2.5
7	22	Female	Monomaxillary	4	2
8	19	Male	Bimaxillary	7	2.4
9	18	Male	Monomaxillary	5	2
10	21	Female	Bimaxillary	6	2.5
11	25	Female	Bimaxillary	7	2.4
12	30	Male	Monomaxillary	8	2
13	27	Female	Bimaxillary	5	2.3
14	26	Female	Bimaxillary	6	2.4
15	19	Female	Monomaxillary	7	1.9
16	20	Male	Bimaxillary	5	2.3
17	19	Male	Monomaxillary	5	2.1
18	19	Female	Bimaxillary	6	2.4
19	20	Female	Bimaxillary	7	2.5
20	21	Female	Bimaxillary	6	2.4

### Surgical outcomes

All 40 mandibular osteotomies and setback procedures were successfully completed without any instances of bad splits. Complete nerve exposure and precise osteotomies were achieved and no reports of permanent anesthesia in the lower lip or chin, and no intraoperative complications related to nerve handling.

### Neurosensory outcomes

Postoperative nerve function recovery was assessed at four intervals: one week, two months, six months, and one year. At one week, all patients (100%) experienced neurosensory disturbances (NSD), including hyperesthesia and hypoesthesia. At two months, NSD decreased to 18 patients (90%). At six months, NSD decreased to 7 patients (35%). At one year, NSD decreased to 1 patient (5%).

**Fig. 8:**
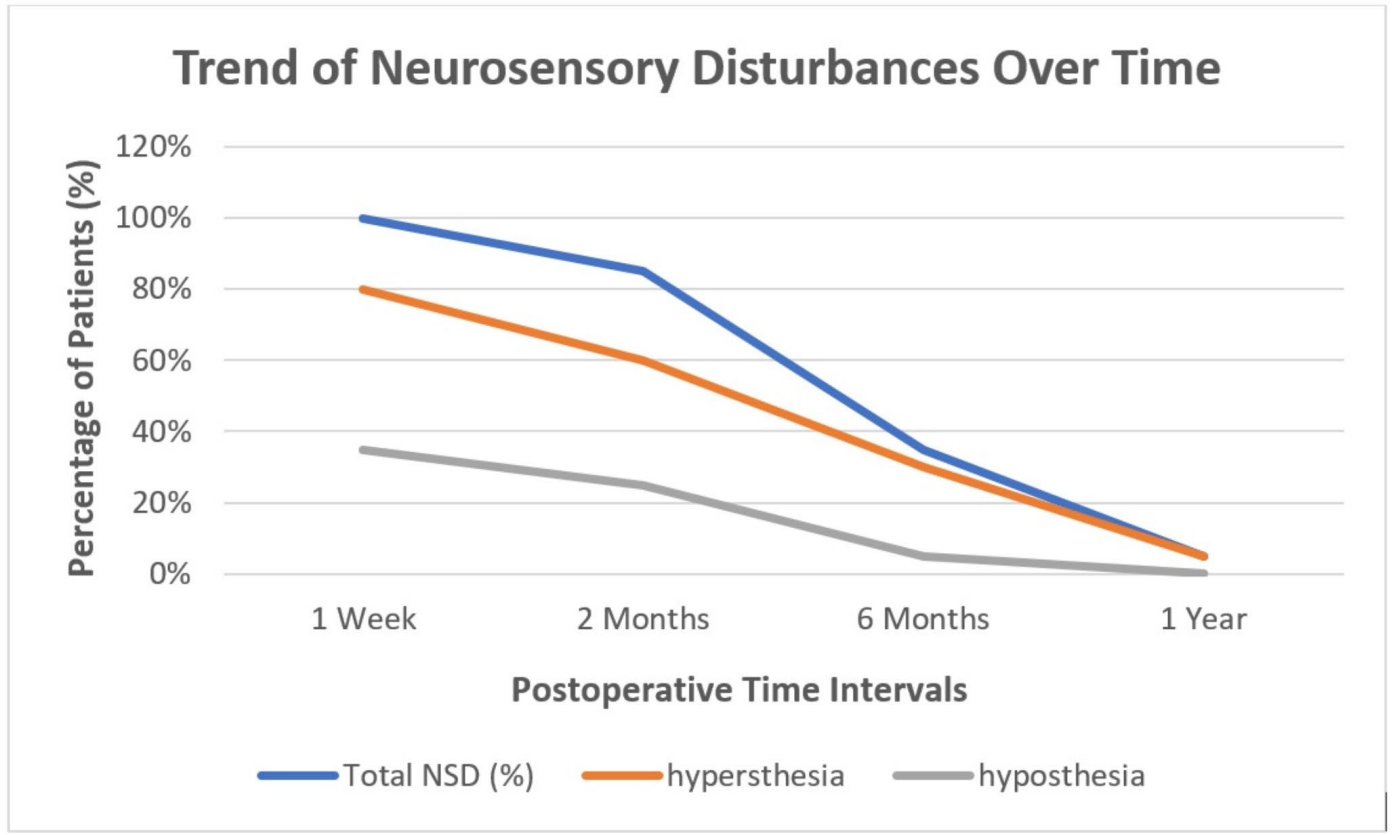
A line graph demonstrating the neurosensory results over time

Hyperesthesia of the Inferior Alveolar Nerve (IAN) showed a progressive decline over the follow-up period. On the right side, hyperesthesia was reported in 15 patients (75%) at one week postoperatively and resolving entirely by one year. On the left side, hyperesthesia was reported in 14 patients (70%) at one week, declining to only one patient (5%) by one year. Similarly, hypoesthesia exhibited a similar recovery pattern. Right IAN hypoesthesia was observed in 5 patients (25%) at one week and resolving completely by six months. Left IAN hypoesthesia was reported in 6 patients (30%) at one week and resolving entirely by one year. (Table 2), (Fig. 8).

**Table 2:** Neurosensory outcomes of the Inferior Alveolar nerve (IAN) at different postoperative intervals

Postoperative follow-up intervals	One week (*n* = 20) No. (%)	Two months (*n* = 20) No. (%)	Six months (*n* = 20) No. (%)	One year (*n* = 20) No. (%)
Total number of patients reported NSD	20 (100)	18 (90)	7 (35)	1 (5)
Right side Hyperesthesia	15 (75)	7 (35)	2 (10)	0 (0)
Left side hyperesthesia	14 (70)	11 (55)	6 (30)	1 (5)
Right side hypoesthesia	5 (25)	2 (10)	0 (0)	0 (0)
Left side Hypoesthesia	6 (30)	3 (15)	1 (5)	0 (0)

## Discussion

Mandibular anatomical variations as rolled-out inferior mandibular border, thin rami with cortically adherent IAN and inferiorly seated IAN canal are considered a critical diagnostic finding which obligates the orthognathic surgeon to select a mandibular osteotomy over another [1]. Conventional mandibular osteotomies introduced in the literature, such as BSSO, ILRO, and C-osteotomy, are utilized for mandibular setback surgery but have notable limitations which direct the authors to find an alternative solution for management of such limitations aiming to reduce the incidence of neurosensory disorders and bad splits along with adequate mandibular setback without distal interferences [2–10]. 

Based on the literature, BSSO is associated with a variable incidence of bad splits, ranging from 0.2 to 14.6%, influenced by anatomical factors such as thin cortices and impacted molars [12, 13]. Moreover, it induces long-term neurosensory disturbances (NSD) which persist in 10–15% of patients at one year [14]. ILRO reduces the risk of nerve injury compared to BSSO but distal interferences limit its application in setback procedures [4]. To address these challenges, Senior et al. introduced a C-osteotomy modification with an intraoral sagittal split below the IAN canal which enhances bone contact, but it remains prone to bad splits and nerve injury in patients with thin buccal cortices (< 7–10 mm), inferiorly positioned IAN or rolled out mandibular border [10]. This directed the authors to designate this new modification for the intraoral ILRO to incorporate nerve lateralization with intra-canal osteotomy.

The incorporation of nerve lateralization ensured safe application of the intra-canal osteotomy and removal of distal interferences with non-blind nerve protection, while the intra-canal osteotomy enhanced the structural integrity of the anterior arm of the proximal segment with 0% incidence of bad splits and adequate rigid intraoral fixation through placement of 4-holes titanium mini-plate.

The fixation method used in this study differs from conventional approaches owing to the novel osteotomy design. Typically, two miniplates with monocortical screws are placed in mandibular body fractures or osteotomies, one in the traction zone and another in the compression zone to enhance stability [15]. However, in the current modified intraoral inverted-L ramus osteotomy, the anterior vertical osteotomy extends from the inferior border and terminates at the level of the inferior alveolar canal, meaning no osteotomy is present at the usual location for the superior plate typically applied in mandibular body osteotomies or fractures. Additionally, the current osteotomy follows a bicortical design, distinct from the traditional BSSO, which may result in different load transmission characteristics [16]. Given these factors, the use of a single miniplate for fixation was deemed appropriate within the scope of this study.

The nerve lateralization of the current osteotomy prompted an evaluation of its NSD risks that relied on subjective assessments such as Shintani Y. et al. who emphasized that subjective evaluations reflect the patient’s experience and provide a more representative outcome compared to objective assessments of neural damage [17]. Abayev et al. reported transient NSD in 99.47% of cases within 1 to 6 months, with permanent NSD in only 0.53% of procedures. Similarly, this study observed transient NSD in 90% of cases, with progressive recovery to 5% at one year [18]. These findings surpass those reported for both BSSO and ILRO, where NSD recovery is slower and incomplete in a significant number of cases [19]. Roychoudhury et al. reported 13.3% NSD for BSSO using subjective assessments after one year follow-up [14]. Moreover, Kobayashi et al. found that NSD rates over one year after ILRO were consistently lower than those for BSSO, with 9.5% vs. 15.2% of cases respectively [20]. In comparison, the current study’s osteotomy achieved only 5% NSD at one year.

Despite the added steps of nerve lateralization and intra-canal osteotomy, the mean surgical duration (2.26 ± 0.21 h) was comparable to traditional methods. The quick nerve exposure time (11 ± 3.2 min per side) and absence of intraoperative complications, such as nerve transection or major bleeding, highlight the technique’s feasibility [21]. Additionally, the use of patient-specific guides through utilizing CBCT imaging and digital planning ensured precise segment separation without unfavorable splits. This is consistent with several studies, that reported improved outcomes with digital integration in orthognathic surgery [11, 22]. 

The combination of preoperative planning and intraoperative guidance minimizes human error and ensures better preservation of anatomical structures [23]. While the study demonstrates promising outcomes, its limitations include a single-center design. Future multicenter studies are necessary to validate these findings. Additionally, comparing this technique directly with other modifications, such as the C-osteotomy or variations of BSSO, would provide more robust evidence of its advantages. Future studies also should compare different fixation techniques to validate optimal fixation methods in relation to this novel osteotomy design and assess its long-term stability.

## Conclusion

The introduced osteotomy overcomes the limitations of the traditional mandibular osteotomies in dealing with mandibular complex anatomy with adequate IAN protection and split segments integrity during mandibular setback surgery.

## Data Availability

No datasets were generated or analysed during the current study.

## References

[1] Aarabi M, Tabrizi R, Hekmat M, Shahidi S, Puzesh A (2014) Relationship between mandibular anatomy and the occurrence of a bad split upon sagittal split osteotomy. J Oral Maxillofac Surg 72(12):2508–2513. 10.1016/j.joms.2014.05.008 25149670 10.1016/j.joms.2014.05.008

[2] Prasad V, Kumar S, Pradhan H, Siddiqui R, Ali I (2021) Bilateral sagittal split osteotomy a versatile approach for correction of facial deformity: a review literature. Natl J Maxillofac Surg 12(1):8–12. 10.4103/njms.NJMS_89_18 10.4103/njms.NJMS_89_18 PMC8191559 34188394

[3] Al-Moraissi EA, Ellis E 3rd (2015) Is there a difference in Stability or neurosensory function between bilateral Sagittal Split Ramus Osteotomy and Intraoral Vertical Ramus Osteotomy for Mandibular Setback? J Oral Maxillofac Surg 73(7):1360–1371. 10.1016/j.joms.2015.01.010Epub 2015 Jan 22. PMID: 25871900 25871900 10.1016/j.joms.2015.01.010

[4] Yamauchi K, Kessler P (2024) The inverted L osteotomy. In: Kessler P, Hardt N, Yamauchi K (eds) Illustrated Manual of Orthognathic surgery. Springer, Cham. 10.1007/978-3-031-06978-9_35.

[5] Kim YK (2017) Complications associated with orthognathic surgery. J Korean Assoc Oral Maxillofac Surg 43(1):3–15. 10.5125/jkaoms.2017.43.1.3Epub 2017 Feb 20. PMID: 28280704; PMCID: PMC5342970 28280704 10.5125/jkaoms.2017.43.1.3 PMC5342970

[6] Jiang N, Wang M, Bi R, Wu G, Zhu S, Liu Y (2021) Risk factors for bad splits during sagittal split ramus osteotomy: a retrospective study of 964 cases. Br J Oral Maxillofac Surg 59(6):678–682 Epub 2020 Aug 28. PMID: 33952406 33952406 10.1016/j.bjoms.2020.08.107

[7] Jędrzejewski M, Smektała T, Sporniak-Tutak K, Olszewski R (2015) Preoperative, intraoperative, and postoperative complications in orthognathic surgery: a systematic review. Clin Oral Investig. 19(5):969– 77. 10.1007/s00784-015-1452-1. Epub 2015 Mar 26. PMID: 25804886; PMCID: PMC4434857 10.1007/s00784-015-1452-1 PMC4434857 25804886

[8] Greaney L, Bhamrah G, Sneddon K, Collyer J (2015) Reinventing the wheel: a modern perspective on the bilateral inverted ‘L’ osteotomy. Int J Oral Maxillofac Surg 44(11):1325–1329. 10.1016/j.ijom.2015.06.003 26183882 10.1016/j.ijom.2015.06.003

[9] Franco PB, Farrell BB (2016) Inverted L osteotomy: a new approach via intraoral access through the advances of virtual surgical planning and custom fixation. Oral Maxillofac Surg Cases 2:1–9. 10.1016/j.omsc.2016.01.001

[10] da Costa Senior O, De Temmerman G, Falter B, Politis C (2021) Modified Intraoral C-Osteotomy. J Craniofac Surg. 32(6):2202–2204. 10.1097/SCS.0000000000007511. PMID: 34516059 10.1097/SCS.0000000000007511 34516059

[11] Omara M, Ali S, Ahmed M (2021) Accuracy of midface advancement using patient-specific surgical guides and pre-bent plates versus conventional interocclusal wafers and conventional plate fixation in quadrangular Le Forte II osteotomy. A randomised controlled trial. Br J Oral Maxillofac Surg 10:S0266. 10.1016/j.bjoms.2021.05.002 10.1016/j.bjoms.2021.05.002 34503857

[12] Verweij JP, Mensink G, Fiocco M, van Merkesteyn JP (2014) Presence of mandibular third molars during bilateral sagittal split osteotomy increases the possibility of bad split but not the risk of other post-operative complications. J Craniomaxillofac Surg. 42(7):e359-63. 10.1016/j.jcms.2014.03.019. Epub 2014 Apr 4. PMID: 24787081 10.1016/j.jcms.2014.03.019 24787081

[13] Steenen SA, Becking AG (2016) Bad splits in bilateral sagittal split osteotomy: systematic review of fracture patterns. Int J Oral Maxillofac Surg 45(7):887–897. 10.1016/j.ijom.2016.02.001 26936377 10.1016/j.ijom.2016.02.001

[14] Roychoudhury S, Nagori SA, Roychoudhury A (2015) Neurosensory disturbance after bilateral sagittal split osteotomy: a retrospective study. J Oral Biol Craniofac Res 5(2):65–68. 10.1016/j.jobcr.2015.04.006Epub 2015 Jun 30. PMID: 26258016; PMCID: PMC4523587 10.1016/j.jobcr.2015.04.006 PMC4523587 26258016

[15] Fonseca RJ (2017) Oral and maxillofacial surgery-inkling enhanced E-Book: oral and maxillofacial surgery-E-Book. Elsevier Health Sciences, p 8

[16] Tseng BT, Yen YC, Cheng CS et al (2022) Biomechanical effects of different miniplate thicknesses and fixation methods Applied in BSSO surgery under two Occlusal conditions. J Med Biol Eng 42:445–458. 10.1007/s40846-022-00733-4

[17] Shintani Y, Nakanishi T, Ueda M, Mizobata N, Tojyo I, Fujita S (2019) Comparison of subjective and objective assessments of neurosensory function after Lingual nerve repair. Med Princ Pract 28(3):231–235. 10.1159/000497610Epub 2019 Feb 6. PMID: 30726857; PMCID: PMC6597906 30726857 10.1159/000497610 PMC6597906

[18] Abayev B, Juodzbalys G (2015) Inferior alveolar nerve lateralization and transposition for Dental Implant Placement. Part II: a systematic review of Neurosensory complications. J Oral Maxillofac Res 6(1):e3. 10.5037/jomr.2014.6103 25937874 10.5037/jomr.2014.6103 PMC4414234

[19] Alolayan AB, Leung YY (2017) Resolution of neurosensory deficit after mandibular orthognathic surgery: a prospective longitudinal study. J Craniomaxillofac Surg 45(5):755–761. 10.1016/j.jcms.2017.01.032Epub 2017 Feb 12. PMID: 28318920 28318920 10.1016/j.jcms.2017.01.032

[20] Kobayashi A, Yoshimasu H, Kobayashi J, Amagasa T (2006) Neurosensory alteration in the lower lip and chin area after orthognathic surgery: bilateral sagittal split osteotomy versus inverted L ramus osteotomy. J Oral Maxillofac Surg 64(5):778–784. 10.1016/j.joms.2006.01.009 16631484 10.1016/j.joms.2006.01.009

[21] Bowe CM, Gurney B, Sloane J, Johnson P, Newlands C (2021) Operative time, length of stay and reoperation rates for orthognathic surgery. Br J Oral Maxillofac Surg 59(2):163–167 Epub 2020 Dec 15. PMID: 33441281 33441281 10.1016/j.bjoms.2020.08.124

[22] Tondin GM, Leal MOCD, Costa ST, Grillo R, Jodas CRP, Teixeira RG (2022) Evaluation of the accuracy of virtual planning in bimaxillary orthognathic surgery: a systematic review. Br J Oral Maxillofac Surg 60(4):412–421 Epub 2021 Sep 20. PMID: 35120785 35120785 10.1016/j.bjoms.2021.09.010

[23] Reddy K, Gharde P, Tayade H, Patil M, Reddy LS, Surya D (2023) Advancements in robotic surgery: a comprehensive overview of current Utilizations and Upcoming Frontiers. Cureus 15(12):e50415. 10.7759/cureus.50415PMID: 38222213; PMCID: PMC10784205 38222213 10.7759/cureus.50415 PMC10784205

